# Detection of Foodborne Pathogens Using Proteomics and Metabolomics-Based Approaches

**DOI:** 10.3389/fmicb.2018.03132

**Published:** 2018-12-18

**Authors:** Snehal R. Jadhav, Rohan M. Shah, Avinash V. Karpe, Paul D. Morrison, Konstantinos Kouremenos, David J. Beale, Enzo A. Palombo

**Affiliations:** ^1^Department of Chemistry and Biotechnology, Swinburne University of Technology, Melbourne, VIC, Australia; ^2^Land and Water, Commonwealth Scientific and Industrial Research Organisation, Brisbane, QLD, Australia; ^3^Australian Centre for Research on Separation Science, RMIT University, Melbourne, VIC, Australia; ^4^Metabolomics Australia, Bio21 Molecular Science and Biotechnology Institute, The University of Melbourne, Melbourne, VIC, Australia

**Keywords:** food safety, mass fingerprint, proteome, metabolomic profiling, biomarkers, chemometrics, statistical discrimination

## Abstract

Considering the short shelf-life of certain food products such as red meat, there is a need for rapid and cost-effective methods for pathogen detection. Routine pathogen testing in food laboratories mostly relies on conventional microbiological methods which involve the use of multiple selective culture media and long incubation periods, often taking up to 7 days for confirmed identifications. The current study investigated the application of omics-based approaches, proteomics using matrix-assisted laser desorption ionization time-of-flight mass spectrometry (MALDI-ToF MS) and metabolomics using gas chromatography-mass spectrometry (GC-MS), for detection of three red meat pathogens – *Listeria monocytogenes*, *Salmonella enterica* and *Escherichia coli* O157:H7. Species-level identification was achieved within 18 h for *S. enterica* and *E. coli* O157:H7 and 30 h for *L. monocytogenes* using MALDI-ToF MS analysis. For the metabolomics approach, metabolites were extracted directly from selective enrichment broth samples containing spiked meat samples (obviating the need for culturing on solid media) and data obtained using GC-MS were analyzed using chemometric methods. Putative biomarkers relating to *L. monocytogenes*, *S. enterica* and *E. coli* O157:H7 were observed within 24, 18, and 12 h, respectively, of inoculating meat samples. Many of the identified metabolites were sugars, fatty acids, amino acids, nucleosides and organic acids. Secondary metabolites such as cadaverine, hydroxymelatonin and 3,4-dihydroxymadelic acid were also observed. The results obtained in this study will assist in the future development of rapid diagnostic tests for these important foodborne pathogens.

## Introduction

Food safety is a major global concern. Many people suffer from illnesses through consumption of food products contaminated by a variety of foodborne pathogens. Bacteria, viruses, and protozoa are mostly responsible for the high mortality and morbidity rates associated with foodborne illnesses (Li and Zhu, 2017). Many food outbreaks due to pathogens such as *Salmonella* spp., *Escherichia coli*, *Shigella* spp., *Listeria monocytogenes* and *Campylobacter jejuni* have been reported in recent years (Sofos, 2008; Marušić, 2011). Increased industrialization and mass production of agricultural products, globalization of the food supply chain and changes in consumer lifestyle and associated variations in food consumption patterns have caused the emergence of new foodborne pathogens or the re-emergence of other pathogens (Pinu, 2016). In addition, food contamination can lead to expensive product recalls (Sofos, 2008). Therefore, it is necessary to have robust, rapid and reliable methods to detect pathogens in different food products.

Routine testing in food laboratories is still largely dependent on traditional identification using time-consuming culture-based techniques (Jasson et al., 2010; Li and Zhu, 2017). On the contrary, immunological or molecular approaches are more rapid and reliable; however, these methods are expensive and labor-intensive (Anderson et al., 2012). There is a need for techniques that enable rapid and early detection of pathogens to ensure enhanced food safety. Early identification and accurate detection of food pathogens will (1) reduce costs of holding products in cold storage while routine testing is conducted, and results are confirmed and (2) mitigate product recalls. Early pathogen detection not only reduces the risk of food outbreaks but also provides greater product assurance. There is scope for developing innovative pathogen detection tools and/or improving current pathogen detection techniques; omics-based approaches such as proteomics, and metabolomics have great potential in food safety research (Bergholz et al., 2014).

In recent years, proteomics-based matrix-assisted laser desorption ionization time-of-flight mass spectrometry (MALDI-ToF MS) has emerged as a tool for microbial identification and diagnostics (Singhal et al., 2015). MALDI-ToF MS is a rapid, sensitive and an inexpensive technique that involves analysis of intact microbial cells or cell extracts. This process has been used for a number of applications, including microbial identification and strain typing (Jadhav et al., 2014, 2015), epidemiological studies (Croxatto et al., 2012), detection of antibiotic resistance (Wolters et al., 2011; Knox et al., 2014), detection of pathogens in blood (Egli et al., 2015), urine (Burillo et al., 2014), food (Vithanage et al., 2014; Vithanage et al., 2017) and water bodies. This technology relies primarily on the characterization of microbes by analyzing the whole cell proteome in a typical mass range *m*/*z* of 2–20 kDa (mainly ribosomal proteins). We previously developed a 30 h detection scheme for *L. monocytogenes* from various food items, such as milk, cantaloupe, and chicken pâté (Jadhav et al., 2014). One of the disadvantages of MALDI-ToF MS is that it is reliant on existing spectral databases of the mass fingerprints of known microbes and is unable to identify new species. In order to establish a customized spectral database, careful quality control is necessary, but this is an elaborate process. Proteomics can, therefore, be complemented with metabolomics to develop a robust and reliable tool for microbial identification and diagnostics.

Metabolomics is another “omics” approach that analyses low molecular weight compounds (metabolites, molecular weight < 1.5 kDa) in a given biological sample. Research in “food metabolomics” has only gained momentum in the last decade. A few studies have applied this approach for the detection of foodborne pathogens (Cevallos-Cevallos et al., 2011; Singh et al., 2011; Beale et al., 2014). It has been mainly used to determine the composition and traceability of foods, food quality and food safety (Pinu, 2015, 2016). Extensive research has been conducted in the area of metabolomics applied to drug discovery (Cisek et al., 2016), disease diagnosis (Kouremenos et al., 2012), biofilms (Beale et al., 2010, 2013), agriculture (Hall and de Maagd, 2014; Lima et al., 2014), toxicology (Hines et al., 2010), natural products discovery (Kim et al., 2010) and nutrition (Heinzmann et al., 2010; Savolainen et al., 2017).

The current study investigated the application of proteomics and metabolomics approaches for the detection of meat pathogens, namely *L. monocytogenes, E. coli* O157:H7 and *S. enterica*. *Salmonella* spp. and *L. monocytogenes* are responsible for *salmonellosis* and *listeriosis*, respectively, while the Shiga toxin-producing *E. coli* O157:H7 can sometimes result in a serious manifestation called hemolytic uremic syndrome (HUS) in susceptible populations (Rhoades et al., 2009). Current methods of pathogen detection generally rely on a multi-step selective enrichment procedure followed by plating on different solid culture media. Confirmation is performed using biochemical tests (Li and Zhu, 2017) or 16S rRNA sequencing. Routine testing of pathogens such as the ones used in the current study usually takes up to 7 days for detection and confirmation. The current study proposes direct detection of pathogens from selective enrichment broths using MALDI-ToF MS (proteomics) and simultaneous identification of a putative biomarkers using gas chromatography-mass spectrometry (GC-MS-based metabolomics). These analyses would save time and cost by eliminating the need to further test pathogen-negative meat samples, thus enabling greater laboratory throughput. This will enhance product assurance and lead to increased consumer confidence.

## Materials and Methods

### Bacterial Strains and Culture Conditions

The bacteria used in this study included *L. monocytogenes* (ACM 98, Australian Collection of Micro-organisms), *S. enterica* subsp. *enterica* serovar Typhimurium (American Type Culture Collection; ATCC 13311), *E. coli* O157:H7 (ATCC 43895) and *E. coli* (ATCC 8739). *E. coli* (ATCC 8739) was used as a reference strain for external calibration of MALDI-ToF MS. Media included brain heart infusion agar (BHIA, Sigma-Aldrich, Australia), Oxoid novel enrichment (ONE) broth for *L. monocytogenes* (OBL, Oxoid, Hampshire, United Kingdom), ONE broth for *Salmonella* (OBS, Oxoid, Hampshire, United Kingdom), Rappaport-Vassiliadis Soya Peptone broth (RVSP, Sigma-Aldrich, NSW, Australia), Muller-Kauffmann tetrathionate broth supplemented with novobiocin (MKTTn, Micromedia, VIC, Australia), *E. coli* medium (EC, Sigma-Aldrich, NSW, Australia), EC supplement with 4-methylumbelliferyl-β-D-glucuronide (EC-Mug, Sigma-Aldrich, NSW, Australia) and tryptic soy broth supplemented with novobiocin (TSBn, Sigma-Aldrich, NSW, Australia). All bacteria were initially grown on BHIA at 37°C, then used for spiking into minced beef samples (purchased from a local supermarket). The spiked foods were enriched in OBL for *L. monocytogenes* or MKTTn for *S. enterica* or TSBn for *E. coli* O157:H7.

Prior to all experiments, a 24 h culture of each pathogen was adjusted to a 0.5 McFarland Standard (equivalent to 1.5 × 10^8^ cfu/mL) in sterile saline using a Vitek colorimeter (Vitek Systems, bioMérieux, Marcy l ‘Etoile, France). This will be referred to as the *standardized culture* in the subsequent sections.

### Spiking Procedure

Minced beef (25 g) was placed in a sterile Stomacher^®^ bag (Seward Limited, Worthing, United Kingdom) with 225 mL of selective enrichment broth. The standardized culture was then added to the beef samples in the sterile filter bag to achieve an initial spiking load of 10 cfu/mL. The spiked samples were homogenized using a Stomacher^®^ 400 laboratory paddle blender (Seward Limited, Worthing, United Kingdom) for 1 min and incubated at 37°C for various time intervals. Aliquots were collected at specified time intervals and subjected to MALDI-ToF MS and GC-MS analysis (see Sections “MALDI-ToF Analysis of Spiked Meat Samples” and “Metabolic Profiling of Spiked Meat Samples Using GC-MS”, respectively).

For process validation, beef samples were screened for the presence of pathogen of interest prior to any further experiments. This was done by enriching the minced beef sample for 24 h in a selective enrichment broth, followed by isolation on pathogen-specific chromogenic agar. Only clean food samples (i.e., those without the pathogen of interest) were used in subsequent experiments.

### MALDI-ToF Analysis of Spiked Meat Samples

To investigate the ability of MALDI-ToF MS to identify bacteria from selective enrichment broth, pilot experiments were performed with different media. The most appropriate broth was used for subsequent experiments.

1. For selective enrichment of *L. monocytogenes*, OBL broth was used according to the method described previously (Jadhav et al., 2014, 2019).
2. For selective enrichment of *S. enterica*, OBS, RVSP and MKTTn broths were used. MKTTn broth was used for further experiments on *S. enterica*.
3. For selective enrichment of *E. coli* O157:H7, EC medium, EC-Mug and TSBn were used. TSBn was used for further experiments on *E. coli* O157: H7.

For detection of *L. monocytogenes*, our previously described 30 h detection scheme (Jadhav et al., 2014) was used. Briefly, a primary enrichment of the spiked meat was performed in OBL broth for 24 h which was followed by a secondary enrichment in OBL for an additional 6 h. Aliquots (1 mL) of the selective enrichment broths were taken at different sampling times for analysis using MALDI-ToF MS.

For detection of *S. enterica*, a single enrichment was performed in MKTTn broth and the broth was sampled at 12 and 18 h. Aliquots (1 mL) of the selective enrichment broths were taken at different sampling times for analysis using MALDI-ToF MS.

In the case of *E. coli* O157:H7, since no single enrichment broth yielded consistent identification, a primary enrichment of the spiked meat was performed in TSBn and 1 mL aliquots were analyzed using MALDI-ToF MS after 12, 18, and 24 h of incubation. Since consistent identifications could not be achieved for any of these conditions, a different scheme was developed. This involved selective enrichment in TSBn for 6 h which was followed by incubation on Rainbow agar for a further 18 h (37°C). Individual colonies showing characteristic morphologies of *E. coli* O157:H7 (as per manufacturer’s instructions) were analyzed using MALDI-ToF MS.

#### Sample Processing for MALDI-ToF MS

The broth aliquots taken for MALDI-ToF MS analysis were processed as per the protocol previously reported (Jadhav et al., 2014). In the case of *E. coli* O157:H7, individual colonies were spotted on the MALDI target plate in triplicate and overlaid with 1 μL CHCA matrix solution. The spots were allowed to air dry prior to analysis.

#### Evaluation of Profiles Using MALDI-ToF MS

Mass spectral peak lists for the test samples were obtained in the mass range of 2 to 20 kDa using the Launchpad software (version 2.9, Shimadzu) and exported to the SARAMIS database (version 4.10) for identification (Jadhav et al., 2014, 2015). The identification in SARAMIS is based on a pattern-matching algorithm wherein the test spectra exported are compared with “SuperSpectra” in the database, resulting in the identification with a confidence score ranging from 75 to 99.9%. In the current study, since well-characterized isolates were used, the identification at the genus or species level was considered as a confirmed identification for confidence scores ≥75%.

When a test spectrum does not find a match to any of the SuperSpectra in the database, occasionally the system uses an in-built functionality to find the closest match to the reference spectra which is referred to as the “comparison” result here. A “comparison” result is a reliable match but not a confirmed identification if the test spectra matches to one or more spectral profiles from a single genus/species in the database. A comparison result can be obtained for confidence scores >70%.

### Metabolic Profiling of Spiked Meat Samples Using GC-MS

Spiking experiments were performed as described in Section“Spiking Procedure”. OBL broth for *L. monocytogenes*, MKTTn broth for *S. enterica* and TSBn broth for *E. coli* O157:H7 were used to enrich the spiked meat samples (10 cfu/mL of final enrichment broth). In the case of *L. monocytogenes*, the enrichment broth was sampled for GC-MS analysis after 18 and 24 h of incubation (37°C). After 24 h of incubation, 1 mL of the enrichment broth was transferred to 9 mL of sterile OBL. This secondary enrichment broth was sampled for GC-MS analysis after a further 6 h of incubation. For experiments with *S. enterica* and *E. coli* O157:H7, broths were sampled after 12, 18, and 24 h of incubation (42°C for MKTTn and 41°C for TSBn). Aliquots (5 mL) were taken at each sampling point. The non-spiked control samples were sampled simultaneously for GC-MS analysis.

#### Sample Processing for GC-MS

Samples for GC-MS were processed to extract metabolites using the protocol given by Ng et al. (2012) with some modifications. Briefly, approximately 40–50 mg of the freeze-dried sample was used for metabolite extraction. Cold absolute methanol (1 mL, HPLC grade, ScharLab, Sentemanat, Spain) containing ^13^C stearic acid (20 μg/mL, Sigma-Aldrich, NSW, Australia) and ^13^C sorbitol (20 μg/mL, HPLC grade, Sigma-Aldrich, NSW, Australia) as internal standards were added to the dried sample. The samples were vortexed for 1 min and centrifuged at 573 *g* for 15 min at 4°C. The extract (50 μL) was dried in an RVC 2-18 CD plus rotational vacuum concentrator (Martin Christ Gefriertrocknungsanlagen GmbH, Osterode am Harz, Germany) at 210 *g* at 40°C. Samples were derivatized using the protocol described by Karpe et al. (2015). The derivatized sample was transferred to a glass vial with a salinized insert for further analysis by GC-MS.

#### GC-MS Analysis

An Agilent 6890 GC oven coupled with a single-quadrupole 5977A MSD (Agilent Technologies, Australia) was used for the GC-MS analysis (Jadhav et al., 2019). Five experimental replicates of independent samples were analyzed for all conditions.

#### Chemometric Analysis of GC-MS Data

The GC-MS data were initially normalized using the internal standard and then analyzed using multivariate data analysis software SIMCA (version 14.1, MKS Umetrics, Sweden). Initially, principal component analysis (PCA) was undertaken, which is an unsupervised approach to find statistically significant differences between datasets. PCA was followed by partial least square discriminate analysis (PLS-DA). PLS-DA is a supervised model that is a regression extension of PCA that takes advantage of metabolite class information, retention time and relative intensity in order to maximize the statistical discrimination between groups of observations, i.e., spiked and control beef samples in this case. To determine the validity of the PCA and PLS-DA models, *R*^2^*X*, *R*^2^*Y*, and *Q*^2^ values were considered. The *R*^2^*X* and *R*^2^*Y* values define a variation between *X* and *Y* variables of various components in the sample set and *Q*^2^ gives predictability of the model (Azizan et al., 2012). In addition, volcano plots were generated using univariate statistical tools (MetaboAnalyst, version 3.0) (Xia et al., 2015; Xia and Wishart, 2016). Volcano plots assist in quick dissemination and identification of statistically significant metabolites based on metabolite fold changes (ratio of metabolites across control and spiked samples) and FDR adjusted *p*-values.

## Results

### Pathogen Detection From Selective Enrichment Broth Using MALDI-ToF MS

*L. monocytogenes* and *S. enterica* were directly detected from selective enrichment broths after specified time intervals. Based on our previous findings, OBL was used for enrichment of *L. monocytogenes*. To identify the most appropriate media for the detection of *S. enterica* using MALDI-ToF MS, OBS, RVSP, and MKTTn media (without beef samples) were evaluated. Poor identification scores were obtained for all broths (data not shown). MKTTn was the only medium that produced species-level identification, albeit with low confidence score. Table 1 summarizes the results obtained for direct detection of *L. monocytogenes* and *S. enterica* from spiked beef samples at various periods of incubation.

**Table 1:** Detection of pathogens directly from selective enrichment broth containing spiked minced beef sample using MALDI-ToF MS.

Pathogen of interest	Spiking load (cfu/mL)	Period of incubation (h)	Level of identification	MALDI-ToF MS identification
*L. monocytogenes*^1^	1	24	Genus	*Listeria*
		30	Genus	*Listeria*
	10	24	Genus	*Listeria*
		30	Species	*L. monocytogenes*
*S. enterica*^2^	1	12	ND	*S. enterica^∗^*
		18	Species	*S. enterica*
	10	12	ND	*S. enterica^∗^*
		18	Species	*S. enterica*
*E. coli*^3^	1	12	ND	No ID^#^
		18	ND	No ID^#^
	10	12	ND	No ID^#^
		18	ND	No ID^#^


*^1^Selective enrichment broth: OBL; ^*2*^selective enrichment broth: MKTTn; ^*3*^selective enrichment broth: TSBn; ^∗^indicates that the sample produced a “comparison” result (The SARAMIS^®^ database only provides an identification above 75% confidence scores. Using an in-built functionality, one can find the closest match to the reference spectra which is referred to as the “comparison” result here. A “comparison” result can be obtained at confidence scores above 70%). ^#^indicates that the sample produced inconsistent identifications, occasionally 77–80% confidence scores were obtained while No Identification (No ID) was recorded on other occasions; ND: no detection.*

The results in Table 1 show that a genus-level identification of *L. monocytogenes* was achieved for all spiked samples irrespective of initial spiking load and period of incubation. Species-level identification was obtained only when the beef sample was spiked at a higher spiking load (10 cfu/mL) after a two-step 30 h incubation. In case of *S. enterica*, a valid species-level identification was achieved only after 18 h of incubation (Table 1). A result is considered “valid” when the confidence score of identification is above 75%. However, a “comparison” result identified presence of *S. enterica* at an initial spiking load of 10 cfu/mL after a 12 h incubation period.

For the detection of *E. coli* O157: H7, three selective enrichment broths, namely EC, EC-mug and TSBn, were evaluated. None of these media yielded consistent identification. Similarly, no identifications were obtained from mass spectra generated from the sample cultured on EC medium. Spectra obtained from samples cultured on EC-Mug and TSBn gave inconsistent identifications, albeit at low confidence values. Despite these results, TSBn was used for further experiments on *E. coli* due to its wide use in several food standard methods such as EN ISO and FDA BAM methods (British Standards Institution, 2001; Feng et al., 2002, 2011) for identification of *E. coli* O157: H7.

An alternate scheme of detection of *E. coli* O157:H7 from beef samples was proposed in the current study. The proposed scheme includes a 6 h enrichment of the spiked beef sample in TSBn at 41°C, followed by incubation on a chromogenic culture medium, such as Rainbow agar, for another 18 h. Rainbow agar supplemented with tellurite gives extra selectivity for *E. coli* O157:H7 (Manafi and Kremsmaier, 2001). The typical black gray colonies obtained for *E. coli* O157:H7 were then analyzed using the direct spotting method. Positive identifications were obtained for the pathogen using this 24 h detection scheme. In the case of a naturally contaminated meat sample, further confirmation of the presence of the Shiga toxin gene would be required using molecular methods.

### Metabolomic Profiling of Pathogens Using GC-MS

**FIGURE 1:**
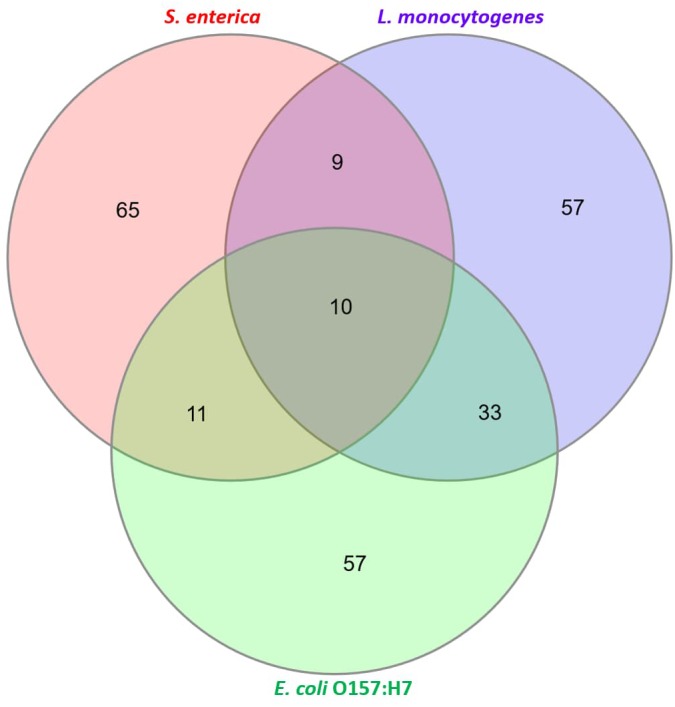
Characterization of total “identified” metabolites. A total of 104 metabolites for *Listeria monocytogenes*, 92 metabolites for *S. enterica* and 104 metabolites for *E. coli* O157:H7 were identified (see Supplementary Information for list of all identified metabolites).

Untargeted metabolomic profiling was performed on control and spiked beef samples collected at various incubation periods. The aim of this exercise was to determine the shortest incubation time required for discriminating the metabolome of spiked samples from that of control samples. The pathogen metabolizes both the substrate (meat) and the broth and, therefore, bacterial metabolites were not separated from the meat and enrichment broth metabolites, i.e., the “system metabolome.” Overall, 501 metabolite features were observed across all samples investigated in this study. A metabolite feature is the disaggregation of each metabolite based on retention time and mass fragment data and does not necessarily represents one metabolite (Beale et al., 2014). A total of 104 metabolites for *L. monocytogenes* contaminated beef samples, 92 metabolites for *S. enterica* contaminated beef samples and 104 metabolites for *E. coli* O157:H7 contaminated beef samples were identified. Figure 1 shows a Venn diagram that illustrates the total “identified” metabolites.

**FIGURE 2:**
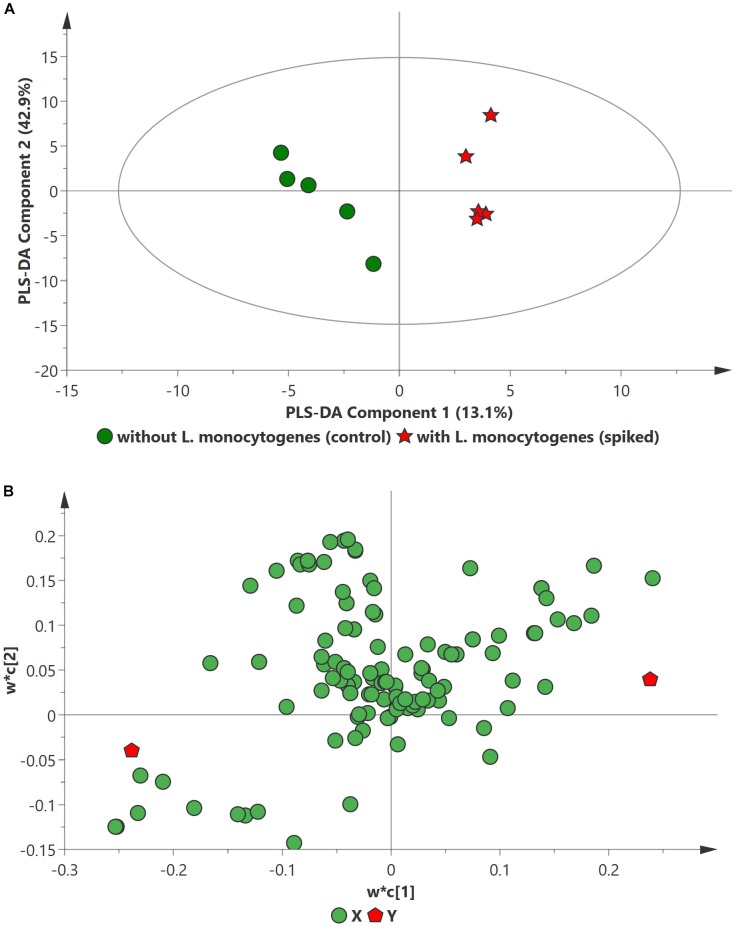
PLS-DA scatter plots of *L. monocytogenes* inoculated in enriched selective media containing beef sample. **(A)** PLS-DA Score scatter plot (*R*^2^*X* = 91.5%, *R*^2^*Y* = 100%, *Q*^2^ = 91.7%). Green circles (•) indicate data obtained from control beef samples and red stars (★) indicate data from spiked beef samples after 24 h of incubation for *L. monocytogenes*. The PLS-DA ellipse (solid line) represents the 95% confidence interval. **(B)** PLS-DA Loading scatter plot.

**FIGURE 3:**
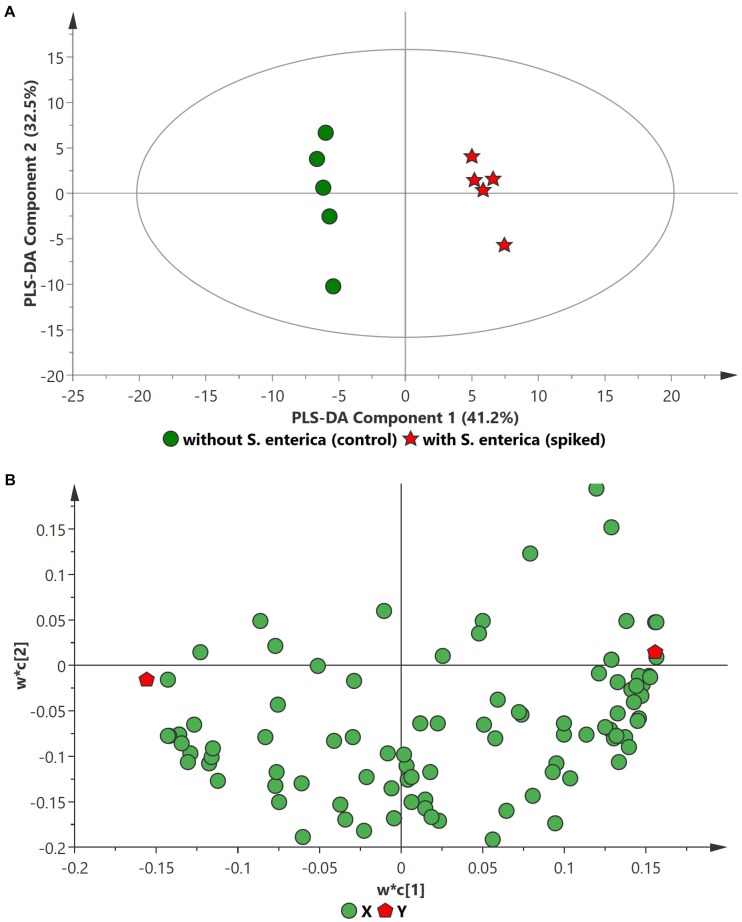
PLS-DA scatter plots of *S. enterica* inoculated in enriched selective media containing beef sample. **(A)** PLS-DA Score scatter plot (*R*^2^*X* = 83.5%, *R*^2^*Y* = 100%, *Q*^2^ = 97.8%). Green circles (•) indicate data obtained from control beef samples and red stars (★) indicate data from spiked beef samples after 18 h of incubation for *S. enterica*. The PLS-DA ellipse (solid line) represents the 95% confidence interval. **(B)** PLS-DA Loading scatter plot.

**FIGURE 4:**
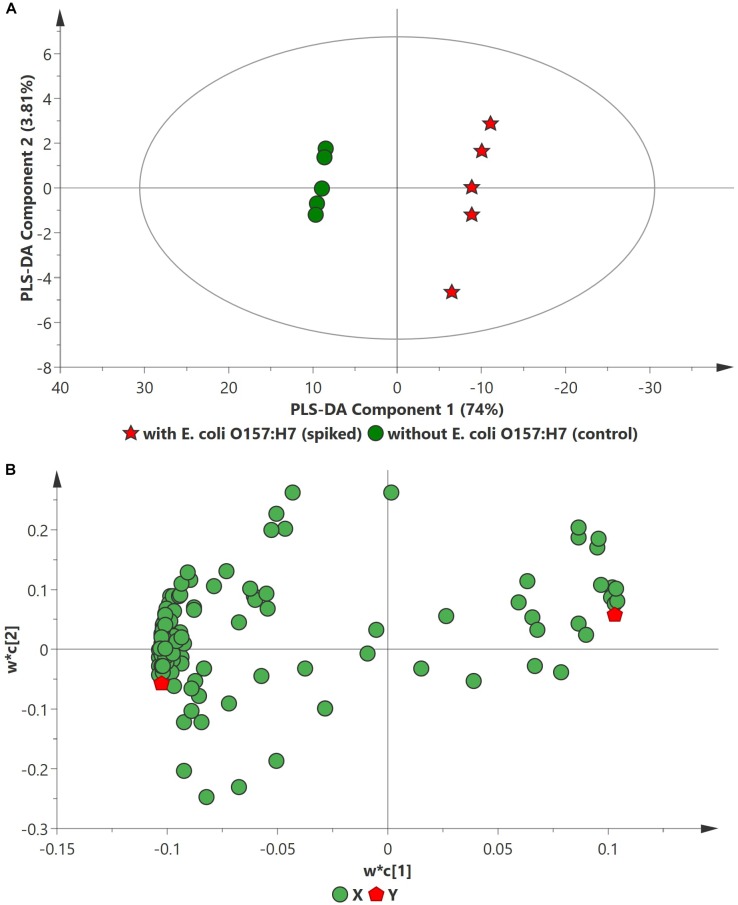
PLS-DA scatter plots of *E. coli* O157:H7 inoculated in enriched selective media containing beef sample. **(A)** PLS-DA Score scatter plot (*R*^2^*X* = 82.8%, *R*^2^*Y* = 100%, *Q*^2^ = 99.3%). Green circles (•) indicate data obtained from control beef samples and red stars (★) indicate data from spiked beef samples after 12 h of incubation for *E. coli* O157:H7. The PLS-DA ellipse (solid line) represents the 95% confidence interval. **(B)** PLS-DA Loading scatter plot.

PCA analysis of inoculated pathogens in beef samples (enriched in selective enrichment medium) showed reasonable discrimination between the spiked and control samples (see Supplementary Information). Due to the unsupervised nature of the data, PCA was observed as a less satisfactory method to discriminate between the metabolite distributions. To strengthen the discrimination between the spiked and control beef samples, PLS-DA analysis was performed on the PCA scatter plot. Figures 2–4 illustrate the PLS-DA scatter plots of inoculated *L. monocytogenes*, *E. coli* O157:H7, and *S. enterica*, respectively, in beef samples. Statistically significant discrimination between the control and spiked samples was obtained at 24 h of incubation for *L. monocytogenes* (Figure 2), 12 h for *E. coli* O157:H7 (Figure 3) and 18 h for *S. enterica* (Figure 4). The *Q*^2^ value for all PLS-DA models were above 90% which is indicative of a model that reasonably fits the data and has a good predictive capability (>70%) (Beale et al., 2017).

**FIGURE 5:**
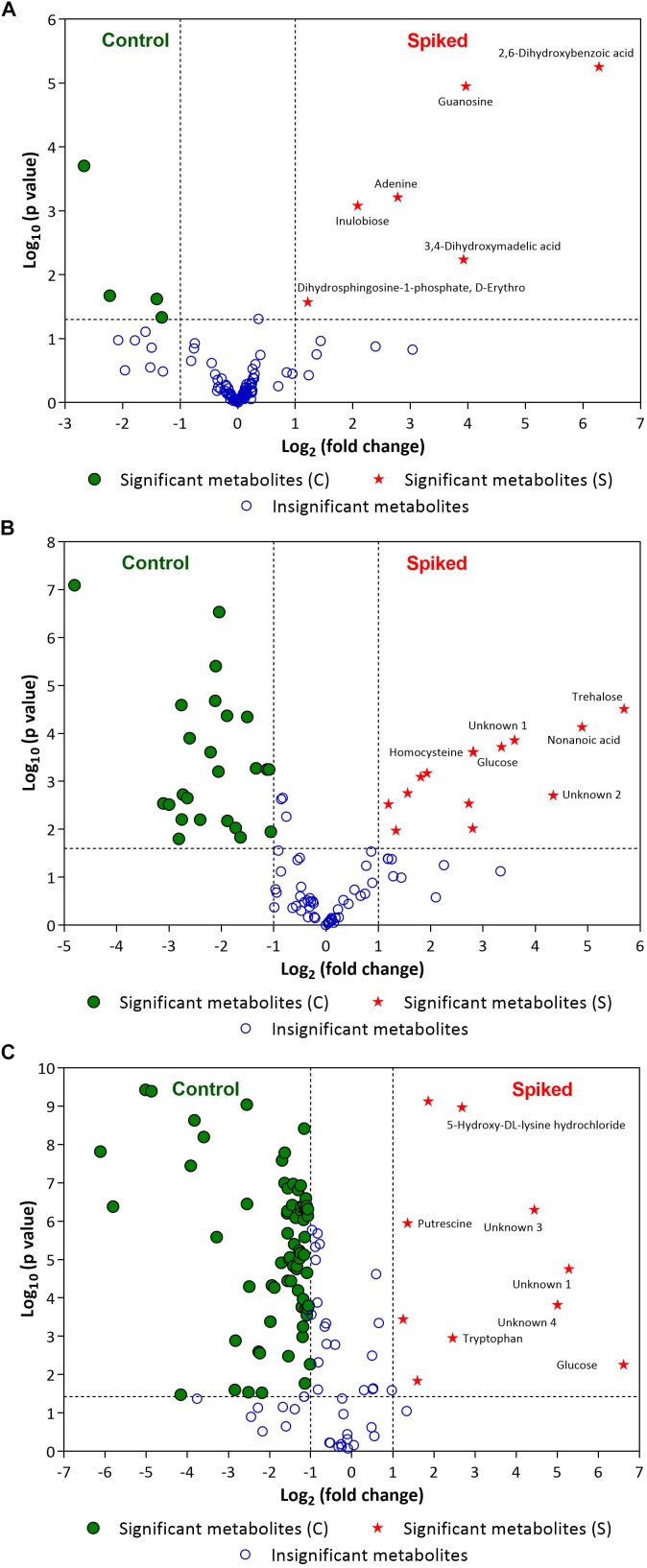
Volcano plots of **(A)** *L. monocytogenes*, **(B)** *S. enterica* and **(C)** *E. coli* O157:H7 enriched in selective media containing the minced beef samples. Green circles (•) and red stars (★) indicate statistically significant putative biomarker metabolites found in control beef samples and spiked meat samples, respectively, after specified period of incubation (For *L. monocytogenes*: 24 h; for *S. enterica*: 18 h and *E. coli* O157:H7: 12 h). A few statistically significant metabolites in spiked samples are labeled in the volcano plots (see Tables 2–4 for top ten statistically significant metabolites). Please refer to Supplementary Information for list of other statistically significant metabolites. Blue open circles (∘) indicate metabolites that are not statistically significant for the discrimination (see Supplementary Information). The dashed lines on the volcano plot represent a *p*-value of 0.05 (*Y*-axis) and a fold change of 2 (*X*-axis).

Figure 5 illustrates the volcano plots of inoculated pathogens in beef samples (against control beef samples). Tables 2–4 list the top ten metabolites for beef samples contaminated with *L. monocytogenes*, *S. enterica*, and *E. coli* O157:H7, respectively, that were identified from the volcano plots.

**Table 2:** Identified significant putative biomarker metabolites (*p*-value < 0.05) in beef sample enriched in OBL and inoculated with *L. monocytogenes*.

Number	Metabolite^1^	Fold change^∗^	*p-*value
1	2,6-Dihydroxybenzoic acid	77.4640	5.60e^-06^
2	Guanosine	15.5800	1.13e^-05^
3	3,4-dihydroxymandelic acid	15.1690	5.82e^-03^
4	Adenine	6.8596	6.16e^-04^
5	Inulobiose	4.2365	8.35e^-04^
6	Erythro-Dihydrosphingosine-1-phosphate	2.3200	2.70e^-02^
7	Glucose	0.1568	1.99e^-04^
8	Arabinose	0.2140	2.13e^-02^
9	Cadaverine	0.3711	2.40e^-02^
10	Uracil	0.4002	4.64e^-02^


*^1^Refer to Supplementary Table S1 for full list of metabolites, ^∗^fold change = 2.0 represents an increase in metabolite concentration, while lower values (anything below FC = 1) represents decrease. An increase of FC = 2 is equivalent to a decrease of FC = 0.5 [on both sides of (0, 0)] on the graph. The metabolites of FC < 0.5 indicate the depleted metabolites and are considered here.*

In case of *L. monocytogenes*, Figures 2, 5A illustrate the PLS DA and volcano plots of the inoculated *L. monocytogenes* minced beef samples, respectively. A total of 104 metabolites were identified across all samples grown in OBL; however, only a small subset, i.e., 10 metabolites, were identified as statistically significant (*p* ≤ 0.05). Table 2 lists the top ten putative biomarker metabolites that resulted in sample discrimination. It is noteworthy that sample discrimination using metabolomics was achieved within 24 h as compared to MALDI-ToF MS which required 30 h for high confidence discrimination. Significantly higher levels (fold change ≥ 2, *p* ≤ 0.05) of 2,6-dihydroxybenzoic acid (fold change ∼77.5), guanosine (fold change ∼15.6), 3,4-dihydroxymadelic acid (fold change ∼15.2), adenine (fold change ∼6.9), inulobiose (fold change ∼4.2) and erythro-dihydrosphingosine-1-phosphate (fold change ∼2.3) were found in spiked beef samples as compared to control samples. In contrast, the levels of glucose (fold change ∼0.16), arabinose (fold change ∼0.21), cadaverine (fold change ∼0.37) and uracil (fold change ∼0.40) were reduced in spiked beef samples. These metabolites could serve as putative biomarkers and form the basis for developing a targeted metabolomics approach for rapid pathogen detection from minced beef samples.

In the case of *S. enterica*, a total of 92 metabolites were identified across all samples enriched in MKTTn. Table 3 lists the top ten putative biomarker metabolites that resulted in sample discrimination. A total of 40 significant putative biomarker metabolites were identified in the inoculated beef samples. The levels of trehalose (fold change ∼51.8), nonanoic acid (fold change ∼29.7), glucose (fold change ∼10.2) and homocysteine (fold change ∼7.0) in spiked beef samples were significantly higher (fold change ≥ 2, *p* ≤ 0.05) than those found in un-inoculated control samples. In contrast, levels of other putative metabolites such as succinic acid (fold change ∼0.04), valine (fold change ∼0.12), butanedioic acid (fold change ∼0.13) and norleucine (fold change ∼0.14) were reduced compared to their control counterparts.

**Table 3:** Identified significant putative biomarker metabolites (*p*-value < 0.05) in beef sample enriched in MKTTn and inoculated with *S. enterica*.

Number	Metabolite^2^	Fold change^∗^	*p*-value
1	Trehalose	51.8240	3.10e^-05^
2	Nonanoic acid	29.6590	7.34e^-05^
3	Unknown 1	20.2530	1.96e^-03^
4	Unknown 2	12.1140	1.39e^-04^
5	Glucose	10.2130	1.92e^-04^
6	Homocysteine	7.0323	2.43e^-04^
7	Succinic acid	0.0357	8.05e^-08^
8	Valine	0.1158	2.88e^-03^
9	Butanedioic acid	0.1250	3.03e^-03^
10	Norleucine	0.1424	1.58e^-02^


*^2^Refer to Supplementary Table S2 for full list of metabolites, ^∗^fold change = 2.0 represents an increase in metabolite concentration, while lower values (anything below FC = 1) represents decrease. An increase of FC = 2 is equivalent to a decrease of FC = 0.5 [on both sides of (0, 0)] on the graph. The metabolites of FC < 0.5 indicate the depleted metabolites and are considered here.*

In the case of *E. coli* O157:H7, A total of 104 metabolites were identified across all samples enriched in TSBn and 43 of these were found to be statistically significant (*p* ≤ 0.05). Table 4 lists the top ten putative biomarker metabolites that resulted in sample discrimination. There was a significant increase (fold change ≥ 2, *p* ≤ 0.05) in the levels of glucose (fold change ∼97.6) with three unknown compounds extracted from inoculated beef samples. A targeted metabolomics study would be useful in identifying the unknown metabolites. Levels of other putative biomarker metabolites, such as 6-hydroxymelatonin (fold change ∼0.01), sophorose (fold change ∼0.02), aspartic acid (fold change ∼0.03), butanedioic acid (fold change ∼0.03), uridine (fold change ∼0.06) and cadaverine (fold change ∼0.07), were significantly reduced (*p* ≤ 0.05).

**Table 4:** Identified significant putative biomarker metabolites (fold change limit of 2, *p*-value < 0.05) in beef sample enriched in TSBn and inoculated with *E. coli* O157:H7.

Number	Metabolite^3^	Fold change^∗^	*p*-value
1	Glucose	97.5820	5.55e^-03^
2	Unknown 3	38.8170	1.76e^-05^
3	Unknown 4	32.1110	1.52e^-04^
4	Unknown 1	21.7020	5.07e^-07^
5	6-Hydroxymelatonin	0.0144	1.52e^-08^
6	Sophorose	0.0179	4.13e^-07^
7	Aspartic acid	0.0309	3.74e^-10^
8	Butanedioic acid	0.0341	4.05e^-10^
9	Uridine	0.0560	3.31e^-02^
10	Cadaverine	0.0662	3.58e^-08^


*^3^Refer to Supplementary Table S3 for full list of metabolites, ^∗^fold change = 2.0 represents an increase in metabolite concentration, while lower values (anything below FC = 1) represents decrease. An increase of FC = 2 is equivalent to a decrease of FC = 0.5 [on both sides of (0, 0)] on the graph. The metabolites of FC < 0.5 indicate the depleted metabolites and are considered here.*

**FIGURE 6:**
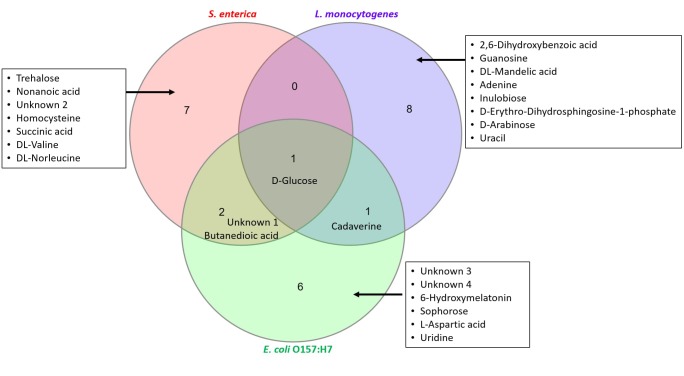
Characterization of “similar” and “unique” metabolites that are statistically significant. Top ten metabolites identified in Tables 2–4 have been used to construct the Venn diagram.

Figure 6 is a Venn diagram that illustrates the number of significant metabolites that are “similar” and “unique” to the investigated pathogens of interest.

## Discussion

Conventional methods of pathogen detection are still widely used in food laboratories. These methods rely heavily on a range of selective media and are known to be time-consuming and cumbersome (Li and Zhu, 2017). The approaches suggested in the current study have the ability to transform routine diagnostics into a more rapid and sensitive process.

Previous studies performed in our laboratory indicate that identification of bacteria from solid culture media using MALDI-ToF MS is a rapid method of detection (Jadhav et al., 2014, 2015). Direct detection of bacteria from selective enrichment broth may attract particular interest given that it may further reduce the time required for testing. The reduced time required for testing will not only apply to food samples contaminated with pathogens but will also apply to pathogen-free food samples, thereby reducing the overall testing time. From our findings, direct detection from selective enrichment broth promises to reduce this time to under 30 h.

Successful identification at genus-level with MALDI-ToF MS was not achieved for beef samples spiked with *L. monocytogenes* at an initial spiking load of 1 cfu/mL after 24 h or 30 h and at 10 cfu/mL after 24 h. A two-step enrichment was required in OBL broth for successful species-level identification at a spiking load of 10 cfu/mL. In the case of *S. enterica*, only “comparison” results produced an identification after 12 h of incubation with the version of the SARAMIS^®^ database used here. These results are consistent with our previous findings on complex foods containing other inherent microbiota (Jadhav et al., 2014). The presence of interfering spectral peaks due to media and meat proteins in the enrichment broth may interfere with the algorithm used for speciation. Thus, a secondary enrichment is deemed necessary to eliminate these interfering peaks. This may explain the inability of SARAMIS to speciate confidently after 30 h from OBL at a lower initial load. Similar findings were reported by Sparbier et al. (2012), where a lower identification rate for *Salmonella* sp. was observed due to the presence of interfering fecal proteins in *Salmonella*-specific enrichment broth. In the case of *E. coli* isolates, none of the selective enrichment broths tested was able to identify the O157:H7 strain with a high confidence score. Therefore, a two-step scheme was considered which involved a 6 h incubation in TSBn followed by an 18 h plating on Rainbow agar. The proposed detection schemes will have to be validated using naturally contaminated meat samples which may contain various inherent microbiota and will be the subject of future research. Overall, with some customization of the database, MALDI-ToF MS seems to be a plausible option for detecting the three meat pathogens investigated in this study.

The proteomics-based approach was complemented with a metabolomics-based approach. Since a whole-cell metabolome approach was used, most of the identified metabolites were sugars, fatty acids, amino acids, nucleobases and organic acids (Figure 1). Secondary metabolites were also observed. Using chemometrics, putative biomarker metabolites were identified that differentiated an artificially spiked meat sample from a control meat sample. These biomarkers could be useful in developing rapid diagnostic tests for foodborne pathogens.

In case of *L. monocytogenes*, 10 statistically significant metabolites were identified (Table 2). Some of these, such as 2,6-dihydroxybenzoic acid and 3,4-dihydroxymadelic acid, are common compounds involved in aminobenzoate degradation pathways (sourced from KEGG database) (Kanehisa and Goto, 2000); however, the actual amount synthesized may vary in different bacteria. Common compounds such as amino acids and cadaverine (generally produced due to putrefaction of animal tissue) were also found in our study, which was in accordance with Cevallos-Cevallos et al. (2011). In comparison with the proteomics approach that required 30 h for a confirmed identification of *Listeria*, the metabolomics approach only required 24 h.

For *S. enterica*, 40 significantly changing metabolites were observed between the control and inoculated samples. While no single putative biomarker was found to be present in the inoculated samples and absent in the control samples, the levels of sugars, such as trehalose and glucose, were found to be significantly different between the two groups. Two unknown compounds were also found in inoculated beef samples at significantly higher levels (*p* ≤ 0.05). A targeted metabolomics protocol using LC-MS or NMR would be required to identity these unknown compounds. The putative biomarker metabolites were identified in 18 h, comparable with MALDI-ToF MS.

In the case of *E.* *coli* O157:H7, 43 metabolites were found to be significantly different between the control and inoculated samples. Unlike MALDI-ToF MS analysis, a different metabolomics detection scheme was not required for *E. coli* O157:H7. Selective enrichment of *E. coli* O157:H7 in TSBn for 12 h, without the requirement of a secondary enrichment, was sufficient for GC-MS based sample differentiation. The levels of sugars such as sophorose and glucose and amino acids such as L-aspartic acid were found to be significantly different between the two groups. Other common compounds, such as cadaverine, butanedioic acid, and uridine were also found to be significantly different between the two groups. Three unknown compounds were found in inoculated beef samples at significantly higher levels (*p* ≤ 0.05).

Figure 6 shows that glucose is a significant metabolite identified in all samples irrespective of contaminating pathogen. Whilst levels of glucose increased significantly in *S. enterica* (fold change ∼10.2) and *E. coli* O157:H7 (fold change ∼97.6), there was a drastic decrease of glucose levels seen in *L. monocytogenes* (fold change ∼0.15). Cadaverine was found in samples contaminated with *L. monocytogenes* and *E. coli* O157:H7. Butanedioic acid and an unknown compound were found in samples contaminated with *S. enterica* and *E. coli* O157:H7. There are several other significant metabolites that are unique to the investigated pathogens of interest (see list of compounds in inset, Figure 6). All these results indicate that a single biomarker metabolite exclusive to a pathogen (qualitative analysis) would be difficult to find. However, by performing a targeted metabolomics study, we can use a combination of compounds to develop rapid diagnostic tests for these pathogens. LC-MS investigation may assist in finding additional biomarker metabolites that can prove to be useful for rapid pathogen detection and will be the subject of future research.

While both the proteomics and metabolomics approaches were found to be suitable for detecting the three pathogens, the authors would like to highlight the importance of controlling the testing conditions in order to generate reproducible results. This includes the precise testing times (due to the dynamically changing metabolites) and, more importantly, standardization of selective media (as observed in the proteomics approach), incubation conditions and sample processing.

## Conclusion

In summary, MALDI-ToF MS proved to be a rapid and reliable method to detect the target pathogens viz. *L. monocytogenes*, *S*. *enterica*, and *E. coli* O157:H7 from meat samples. Direct detection schemes for *L. monocytogenes* and *S. enterica* isolates from selective enrichment broth (avoiding the need for an additional step of culturing on solid media) using MALDI-ToF MS were proposed. The proposed methodologies can significantly reduce the time required for detecting these three pathogens. The proposed methodology will have to be further validated by testing naturally contaminated meat samples. Another mass spectrometry-based platform viz. GC-MS was used to identify potential biomarker metabolites from the complex matrix (beef samples) to identify contamination by the target pathogens. Since an untargeted metabolomics approach was used in this study, a large number of unknown metabolites were found with a few being statistically significant. A targeted approach will be performed in future studies to assist in the development of rapid diagnostic tests for these important meat pathogens.

## Author Contributions

SJ designed the experiments, performed the experimentation, wrote the manuscript, and analyzed the data. RS wrote the manuscript and analyzed the data. AK analyzed the data. PM performed the experimentation. DB, KK, and EP designed the experiments and wrote the manuscript.

## Conflict of Interest Statement

The authors declare that the research was conducted in the absence of any commercial or financial relationships that could be construed as a potential conflict of interest.
