# Supplementary material for: Detection of Foodborne Pathogens Using Proteomics and Metabolomics-Based Approaches
Source: Front Microbiol. 2018 Dec 18;9:3132. doi: 10.3389/fmicb.2018.03132 (PMC6305589; doi:10.3389/fmicb.2018.03132)
Supplement: Supplementary file 1 [file Data_Sheet_1.pdf]

Detection of foodborne pathogens using a whole-cell enrichment proteomics and metabolomics-based approach

Snehal R. Jadhav [1#], Rohan M. Shah [1], Avinash V. Karpe [1, 2], Paul D. Morrison [3], Konstantinos Kouremenos [4], David J. Beale [2] and Enzo A. Palombo [1, *]

[1] Department of Chemistry and Biotechnology, Swinburne University of Technology, Melbourne, Australia

[2] Land and Water, Commonwealth Scientific and Industrial Research Organisation (CSIRO), Brisbane, Australia

[3] Australian Centre for Research on Separation Science, RMIT University, Melbourne, Victoria, Australia

[4] Metabolomics Australia, Bio21 Molecular Science and Biotechnology Institute, The University of Melbourne, Melbourne, Australia

[*]Corresponding author email:

epalombo@swin.edu.au (EAP)

# Current address: Centre for Advanced Sensory Science, School of Exercise and Nutrition Sciences, Deakin University, Melbourne, Australia (snehal.jadhav@deakin.edu.au)

Supplementary Information

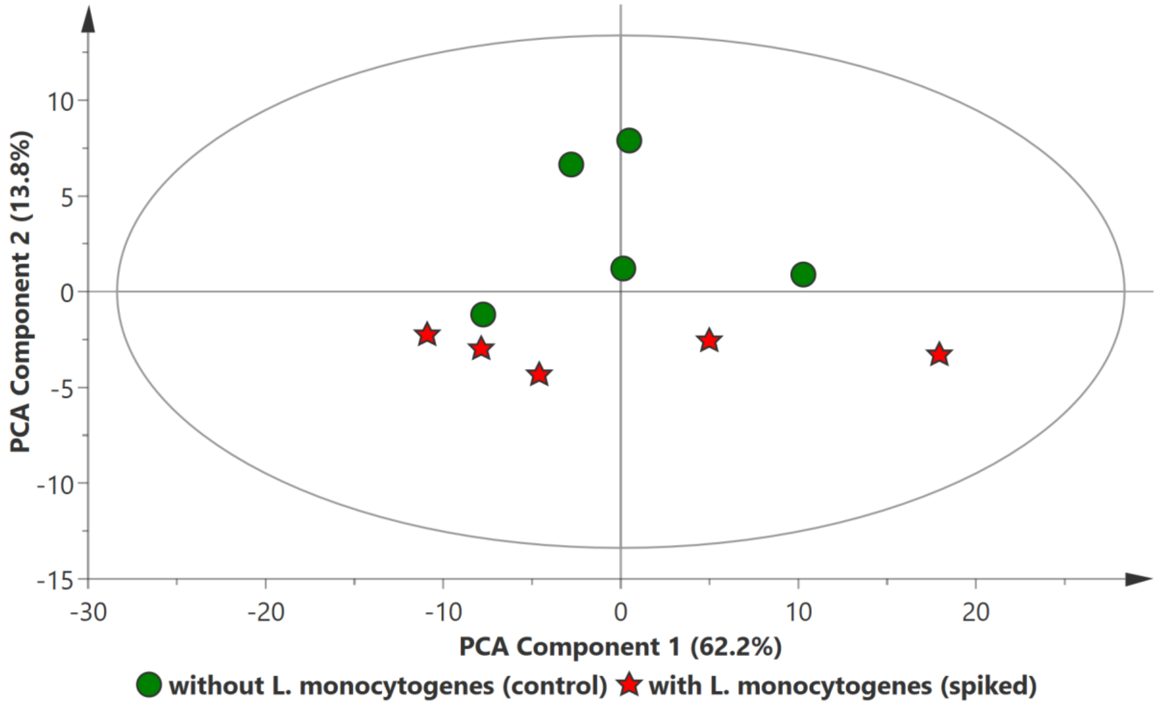
PCA Component 2 (13.8%)
PCA Component 1 (62.2%)
without L. monocytogenes (control)
with L. monocytogenes (spiked)

(A)

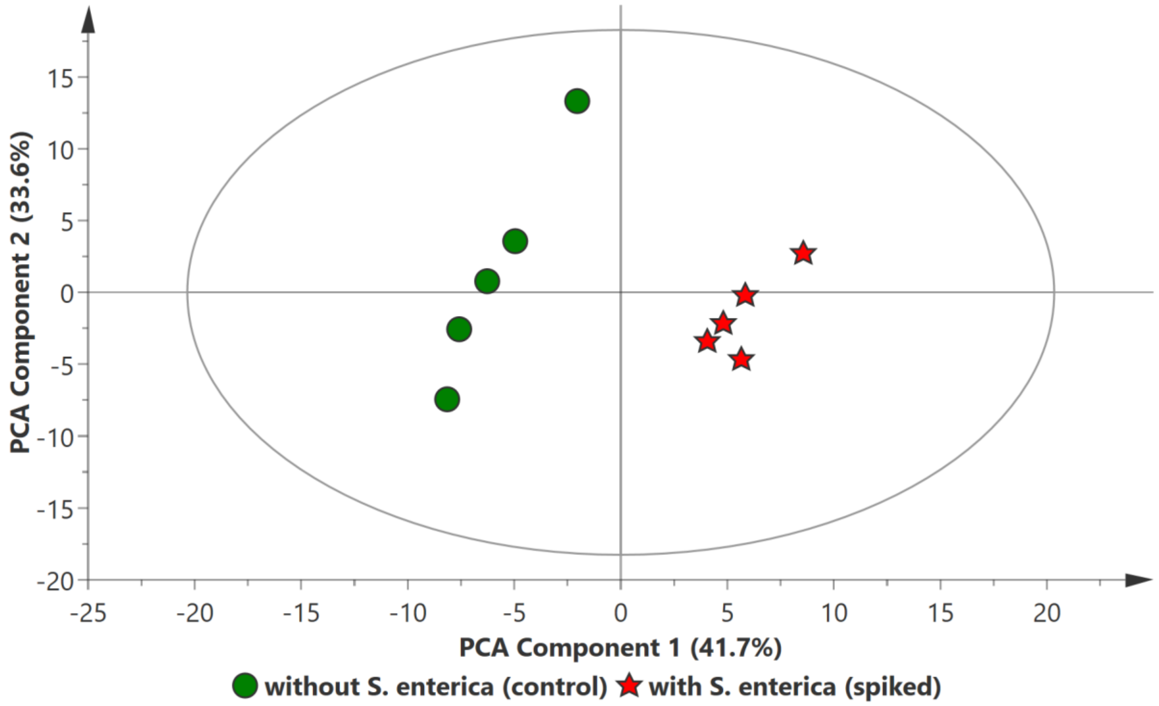
PCA Component 2 (33.6%)
PCA Component 1 (41.7%)
without S. enterica (control)
with S. enterica (spiked)

(B)

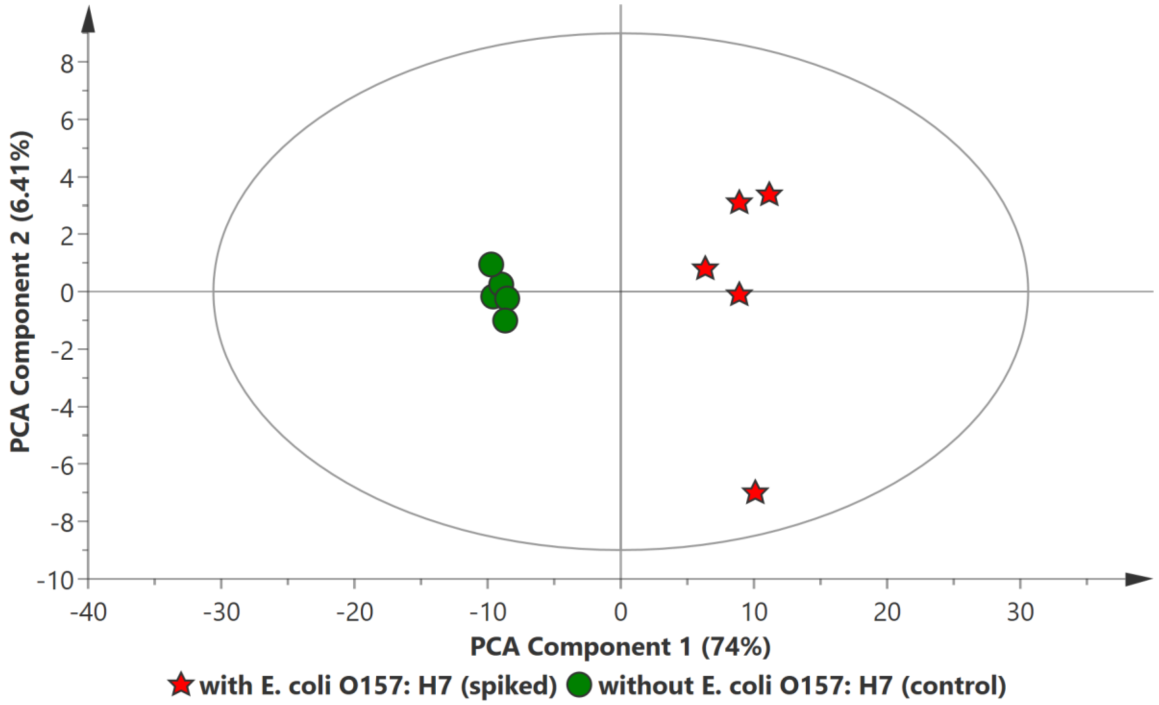

(C)

Figure S1. PCA scatter plots of (A) *Listeria monocytogenes* (R^2X=85% and Q^2= 64.7%)**, (B) *Salmonella enterica*** (R^2X=87.7% and Q^2= 48.1%) **and (C) *Escherichia coli* O157:H7** (R^2X=80.4% and Q^2= 63.2%) **enriched in selective media containing the minced beef samples.** Green circles (●) indicate data obtained from control beef samples and red stars (★) indicate data from spiked beef samples after specified period of incubation (For *L. monocytogenes*: 24 h; for *S. enterica*: 18 h and *E. coli* O157:H7: 12 h). The PCA ellipse (solid line) represents the 95% confidence interval.

Table S1. Identified metabolites in *L. monocytogenes*-spiked beef samples

Number	Metabolite	Fold change	P value
1	2,6-dihydroxybenzoic acid	77.4640	5.60E-06
2	Guanosine	15.5800	1.13E-05
3	3,4-dihydroxymadelic acid	15.1690	5.82E-03
4	2,4,6-Tris(trimethylsiloxy)-3-pyridincarbonitrile	8.1919	1.48E-01
5	Adenine	6.8596	6.16E-04
6	Fructose	5.2500	1.33E-01
7	Inulobiose	4.2365	8.35E-04
8	Trehalose	2.7127	1.09E-01
9	Acetylenedicarboxylic acid	2.5806	1.77E-01
10	Gluconic acid	2.3504	3.73E-01
11	Dihydrosphingosine-1-phosphate, D-Erythro	2.3200	2.70E-02
12	Xylitol	1.9308	3.52E-01
13	Compound_78	1.8001	3.37E-01
14	Ribose-5-phosphate	1.6279	5.56E-01
15	N-acetyl-D-galactosamine	1.2829	4.92E-02
16	Sophorose	1.2359	2.51E-01
17	Maleamic acid	1.2249	3.57E-01
18	Hexadecanoic acid	1.2154	4.21E-01
19	Fructose-6-phosphate	1.2005	4.11E-01
20	Glucose-6-phosphate	1.2004	4.11E-01
21	Tryptophan	1.1922	2.97E-01
22	Citric acid	1.1915	6.43E-01
23	Octadecanoic acid	1.1861	5.11E-01
24	Compound_112	1.1856	6.62E-01
25	Pentanoic acid	1.1855	6.98E-01
26	Ribitol	1.1799	5.86E-01
27	Histidine	1.1763	5.00E-01
28	6-Hydroxymelatonin	1.1746	6.72E-01
29	1-phenyl-1-(2-trimethylsilylcyclopentyl)ethanol	1.1736	6.73E-01
30	Ornithine-lactam	1.1725	5.20E-01
31	Phosphoric acid	1.1721	8.82E-01
32	2-phenylsulphonyl-2-trimethylsilylpropane	1.1451	4.99E-01
33	Norepinephrine	1.1201	7.30E-01
34	3-[(trimethylsilyl)oxy]indene	1.1143	7.52E-01
35	Sphingosine	1.1126	7.57E-01
36	Proline	1.1090	5.16E-01
37	Lysine	1.1023	6.53E-01
38	pro-gly	1.0952	6.75E-01
39	Glycine	1.0915	5.90E-01
40	Mandelic acid	1.0871	6.47E-01
41	Threo-beta-hydroxyaspartic acid	1.0871	6.47E-01
42	Methyl-L-serine	1.0861	6.52E-01
43	Homoserine	1.0848	6.63E-01

Number	Metabolite	Fold change	P value
44	5-Hydroxytryptophan	1.0830	6.70E-01
45	Isoleucine	1.0829	8.72E-01
46	Glutamic acid	1.0798	7.20E-01
47	Phenylalanine	1.0787	6.76E-01
48	12-Crown-4	1.0714	8.19E-01
49	3,5-Diiodo-L-Tyrosine	1.0701	7.20E-01
50	Ribose	1.0623	7.26E-01
51	Asparagine	1.0610	7.33E-01
52	Dihydroxyflavan-4-ol	1.0395	8.55E-01
53	Serine	1.0387	8.44E-01
54	Mannitol	1.0300	8.42E-01
55	Erythritol	1.0271	8.59E-01
56	Tyrosine	1.0242	8.95E-01
57	2-oxo-Glutaric acid	1.0185	9.27E-01
58	Aspartic acid	1.0179	9.28E-01
59	Glyceric acid	1.0157	9.44E-01
60	Propanoic acid	1.0157	9.44E-01
61	Carnosine	1.0124	9.68E-01
62	Pipecolic acid	1.0121	9.71E-01
63	Pyroglutamic acid	1.0121	9.71E-01
64	Threonine	1.0104	9.58E-01
65	Butanedioic acid	1.0033	9.89E-01
66	Succinic acid	1.0033	9.89E-01
67	Oxalic acid	0.9923	9.71E-01
68	Oxamic acid	0.9913	9.68E-01
69	Acetylcholine chloride	0.9863	9.56E-01
70	Compound_59	0.9807	9.55E-01
71	Glycerol	0.9755	9.45E-01
72	Urea	0.9531	8.75E-01
73	Adenosine-3'-Monophosphoric acid	0.9467	9.34E-01
74	3-Oxalomalate	0.9328	8.02E-01
75	Beta-Alanine	0.9275	8.20E-01
76	Aminooxyacetic acid	0.9227	7.74E-01
77	Glycerol-3-phosphate	0.9179	7.24E-01
78	Ornithine	0.9166	7.60E-01
79	Adenosine	0.9131	8.89E-01
80	Compound_49	0.8958	7.16E-01
81	Compound_130	0.8950	7.57E-01
82	Pyruvic acid	0.8921	5.99E-01
83	Lactic acid	0.8752	5.55E-01
84	myo-inositol	0.8748	7.22E-01
85	Glycolic acid	0.8744	5.54E-01
86	Butanal, 2,3,4-tris[(trimethylsilyl)oxy]-3-[[(trimethylsilyl)oxy]methyl]-, O-methyloxime, (S)-	0.8510	6.43E-01

Number	Metabolite	Fold change	P value
87	1,8-Diaminooctane	0.8224	4.22E-01
88	5-Hydroxy-DL-lysine hydrochloride	0.8217	4.21E-01
89	Norleucine	0.8110	6.09E-01
90	Compound_119	0.7892	5.74E-01
91	2,3,4-Trihydroxybutyric acid	0.7600	3.60E-01
92	Methionine	0.7302	2.40E-01
93	2,2-Dimethylsuccinic acid	0.5954	1.18E-01
94	4-[bis(trimethylsilyl)amino]-butanoic acid	0.5954	1.18E-01
95	Propanedioic acid	0.5954	1.18E-01
96	Cysteine sulfate	0.5895	1.42E-01
97	Glutamine	0.5694	2.23E-01
98	Uracil	0.4002	4.64E-02
99	Cadaverine	0.3771	2.40E-02
100	Benzoic acid	0.3486	2.82E-01
101	Allo-inositol	0.3285	7.79E-02
102	Lithocholic acid	0.2892	1.07E-01
103	Arabinose	0.2140	2.13E-02
104	Glucose	0.1568	1.99E-04

Table S2. Identified metabolites in *S. enterica*-spiked beef samples

Number	Metabolite	Fold change	P value
1	Trehalose	51.824	3.10E-05
2	Nonanoic acid	29.659	7.34E-05
3	Compound_63	20.253	1.96E-03
4	Compound_25	12.114	1.39E-04
5	Glucose	10.213	1.92E-04
6	Leucine	10.081	7.48E-02
7	Homocysteine	7.0323	2.43E-04
8	2'-Deoxyuridine	7.0078	2.45E-04
9	3-Butyn-1-ol	7.0078	2.45E-04
10	3-(trimethylsilyl)-1-(tert-butyldimethylsiloxy)-4-pentene	6.9646	9.56E-03
11	Compound_16	6.6297	2.89E-03
12	cis-1-methyl-2-trimethylsilylmethylcyclo-hexan-1-ol	4.7651	5.59E-02
13	2,6-Dihydroxybenzoic acid	4.2723	2.61E-01
14	Propanoic acid, 2-methyl-2,3-bis[(trimethylsilyl)oxy]-, trimethylsilyl ester	3.8048	6.77E-04
15	Tris-{[(tert-butyl)dimethylsilyl]oxy}cyclohex-2-en-1-one	3.5022	8.06E-04
16	Glycerol	2.947	1.75E-03
17	Compound_106	2.7061	1.01E-01
18	Cysteine sulfate	2.5196	1.07E-02
19	Nonanoic acid	2.4302	9.48E-02
20	Propanoic acid	2.3828	4.20E-02
21	Ribose	2.2821	3.01E-03
22	N,O-Bis-(trimethylsilyl)-2-pyrrolidone carboxylic acid	2.2734	4.10E-02
23	N-Carbamyl-L-Glutamic acid	2.2731	4.09E-02
24	Fumaric acid	2.2645	4.06E-02
25	Lyxosylamine	1.8449	1.30E-01
26	Compound_96	1.6994	5.71E-02
27	Compound_83	1.5782	2.44E-01
28	Pentanoic acid	1.3467	3.60E-01
29	Phosphoric acid	1.2567	2.97E-01
30	Compound_102	1.1828	6.84E-01
31	Heptadecanoic acid	1.171	4.70E-01
32	Compound_103	1.1323	7.04E-01
33	Glycocholic acid	1.1323	7.04E-01
34	Compound_78	1.1061	8.84E-01
35	Shikimic acid	1.0763	8.07E-01
36	Linoleic acid	1.0756	7.10E-01
37	Oleic acid	1.0622	7.59E-01
38	Compound_90	1.0451	8.48E-01
39	Fructose	1.035	8.40E-01

Number	Metabolite	Fold change	P value
40	Hexadecanoic acid	1.0297	8.80E-01
41	Acetyloleanolic acid	1.0259	9.17E-01
42	Compound_105	0.99759	9.90E-01
43	1,2-Bis(trimethylsilyoxy)-4-carbomethoxy-5-methyl-1-cyclohexene	0.86553	7.11E-01
44	Lactic acid	0.85399	6.67E-01
45	1-phenyl-1-phenylsulphonyl-1-trimethylsilylethane	0.84646	3.48E-01
46	Tetradecanoic acid	0.83797	3.18E-01
47	Propanoic acid, 2-methyl-, propyl ester	0.81531	3.21E-01
48	3-dehydroquinate	0.80499	2.71E-01
49	3-Deoxy-arabino-hexonic acid lactone	0.80406	4.13E-01
50	Sulfamic acid	0.78639	6.79E-01
51	Compound_107	0.7837	3.07E-01
52	3-methyl-2-butenyl (E)-2-methyl-3-trimethylsiloxy-2-butenoate	0.74932	3.30E-01
53	5-((2-methoxyethoxy)methoxy)-7-phenyl-2-(trimethylsilyl)-1-heptene	0.71897	5.00E-01
54	Oxamic acid	0.7175	1.58E-01
55	Bis[(1,1-dimethylpropyl)(trimethylsilyl)amino]sulfan	0.71004	2.50E-01
56	Shikimic acid-3-phosphate	0.70536	3.93E-02
57	Cholesterol	0.6841	4.33E-02
58	Compound_58	0.67263	3.97E-01
59	Pyruvic acid	0.64102	4.36E-01
60	N-Glycolylneuraminic acid	0.59139	5.41E-03
61	4-Hydroperoxy-5-decanol	0.56305	2.21E-03
62	2,3-Dimethylsuccinic acid	0.56305	2.21E-03
63	2,4-Dihydroxybutanoic acid	0.55187	2.35E-03
64	1-(phenylthio)-1-(trimethylsilyl)-4-pentanone	0.54994	7.52E-02
65	alpha-ketoisocaproic acid sodium salt	0.53027	2.76E-02
66	4-Hydroxy-3-methoxy-4-(2-propenyl)-2-[(trimethylsilyl)ethynyl]-2-cyclobuten-1-one	0.51828	2.07E-01
67	Compound_104	0.51102	1.78E-01
68	Sodium phenylpyruvate	0.50284	4.24E-01
69	Dodecanoic acid	0.48022	1.13E-02
70	alpha-D-Glucose-1-phosphate, dipotassium salt dihydrate	0.4704	5.60E-04
71	Allo-inositol	0.45668	5.64E-04
72	Uracil	0.39535	5.31E-04
73	Compound_64	0.35215	4.51E-05
74	Sarcosine	0.32171	1.46E-02
75	Phenyllactic acid	0.30173	9.35E-03
76	2-phenylsulphonyl-2-trimethylsilylpropane	0.27071	6.64E-03
77	Proline	0.26903	4.28E-05
78	Nicotinic acid	0.2429	2.92E-07

Number	Metabolite	Fold change	P value
79	alpha-keto-gamma-methiolbutyric acid sodium salt	0.24012	6.24E-04
80	Hexanoic acid	0.23206	3.94E-06
81	2-Butenedioic acid	0.23012	2.09E-05
82	Mannose	0.21621	2.46E-04
83	Compound_95	0.18883	6.30E-03
84	Compound_70	0.16425	1.25E-04
85	Compound_35	0.15923	2.23E-03
86	Propanoic acid, 3-(trimethylsilyl)-, methyl ester	0.15	1.88E-03
87	Glycine	0.14792	6.24E-03
88	2-(methoxyimino)-Butanoic acid	0.14694	2.55E-05
89	Norleucine	0.14239	1.58E-02
90	Butanedioic acid	0.12495	3.03E-03
91	Valine	0.11585	2.88E-03
92	Succinic acid	0.035714	8.05E-08

Table S3. Identified metabolites in *E. coli* O157:H7-spiked beef samples

Number	Metabolite	Fold change	P value
1	Glucose	97.582	5.55E-03
2	Compound_49	38.817	1.76E-05
3	Compound_70	32.111	1.52E-04
4	Compound_63	21.702	5.07E-07
5	5-Hydroxy-DL-lysine hydrochloride	6.4111	1.08E-09
6	Tryptophan	5.4774	1.11E-03
7	Compound_102	3.6291	7.49E-10
8	Compound_84	3.0184	1.45E-02
9	Putrescine	2.5557	1.13E-06
10	Compound_65	2.5218	8.85E-02
11	N-acetyl-D-galactosamine	2.3746	3.62E-04
12	Cellobiose	1.9605	2.55E-02
13	Lysine	1.5067	2.39E-05
14	Succinic acid	1.4641	4.00E-01
15	Lyxosylamine	1.4331	2.27E-02
16	Heptadecanoic acid	1.4065	3.17E-03
17	Glucose-6-phosphate	1.3941	2.37E-01
18	Uracil	1.0352	6.89E-01
19	Compound_118	0.93603	8.36E-01
20	Glycerol-3-phosphate	0.92668	4.82E-01
21	Leucylglycylglycine	0.92666	3.59E-01
22	Dihydrouracil	0.86871	1.07E-01
23	Compound_48	0.84792	4.19E-02
24	γ-Amino-n-Butyric Acid	0.83668	6.48E-01
25	Compound_17	0.79851	7.66E-01
26	2-amino-2-methyl-1,3-propanediol	0.75401	1.66E-03
27	Fructose	0.69442	6.02E-01
28	Xylose	0.68587	5.93E-01
29	Xanthine	0.65271	1.58E-03
30	Glutamic acid	0.64628	4.61E-04
31	Tyrosine	0.63303	5.62E-04
32	Pyroglutamic acid	0.5816	3.89E-06
33	Maleamic acid	0.56429	2.43E-02
34	Proline	0.55975	1.31E-04
35	Isoleucine	0.53954	4.67E-06
36	Valine	0.50599	2.70E-04
37	Compound_60	0.49479	5.33E-03
38	3-(Diethoxymethyl)-1,5-bis(trimethylsilyl)-1,4-pentadiyn-3-ol	0.48271	1.59E-04
39	Leucine	0.4763	1.90E-04
40	Phosphoric acid	0.47628	7.21E-07
41	Glycerol	0.47627	7.20E-07
42	Idose	0.4691	2.20E-05
43	Sarcosine	0.46836	2.82E-04

Number	Metabolite	Fold change	P value
44	Epicatechin	0.46637	4.01E-07
45	Compound_113	0.46058	4.42E-07
46	Propanoic acid	0.45397	1.69E-02
47	2,4-dihydroxybutanoic acid	0.45178	2.59E-06
48	Phenylalanine	0.44825	3.76E-02
49	Hexadecanoic acid	0.44705	3.81E-09
50	3-Oxalomalate	0.44548	7.46E-06
51	Threonine	0.44505	3.86E-07
52	Compound_19	0.43855	5.62E-04
53	Serine	0.43594	1.02E-03
54	Hexanoic acid	0.43078	1.73E-04
55	Compound_97	0.4267	4.16E-07
56	Compound_67	0.42251	5.12E-07
57	Compound_21	0.42249	1.16E-07
58	Myo-inositol	0.42125	6.83E-06
59	Compound_111	0.41502	9.25E-06
60	6-(trimethylsilyl)-5-hexyn-1-ol	0.41317	5.83E-06
61	Pipecolic acid	0.40543	1.53E-07
62	Lactic acid	0.40326	6.35E-05
63	Histidine	0.39557	1.48E-05
64	Methionine	0.38783	8.06E-07
65	Norleucine	0.38171	7.93E-02
66	Oxalic acid	0.37773	3.96E-06
67	Homoserine	0.37329	1.47E-05
68	Sucrose	0.37222	1.06E-07
69	Urea	0.36679	3.78E-07
70	Compound_106	0.3585	3.59E-05
71	Ornithine	0.35254	8.73E-06
72	Compound_120	0.34853	9.61E-06
73	Glycine	0.33993	1.38E-07
74	Compound_126	0.33938	3.56E-05
75	pro-gly	0.33901	2.05E-06
76	Citric acid	0.33802	5.43E-07
77	Compound_108	0.33693	6.23E-07
78	Valero-1,5-lactam	0.32885	2.23E-01
79	Compound_122	0.32234	1.63E-08
80	Compound_100	0.32175	1.00E-07
81	Alpha-D-methyl-galactoside	0.30827	2.59E-08
82	Sinapyl alcohol	0.30494	1.21E-05
83	3-Amino-2-piperidone	0.27087	5.34E-05
84	(2-hydroxyethyl)-beta-Alanine	0.26001	4.71E-05
85	Compound_89	0.2532	4.16E-04
86	Beta-Alanine	0.22188	2.99E-01
87	Compound_125	0.21998	2.96E-02
88	Carnosine	0.21204	2.73E-03

Number	Metabolite	Fold change	P value
89	5-Methoxy-Tryptamin	0.20769	2.54E-03
90	Compound_110	0.20513	7.32E-02
91	Lumichrome	0.18277	1.25E-01
92	2-amino-butyric acid	0.17746	5.04E-05
93	Triethylamine, 2-(trimethylsiloxy)	0.17073	3.54E-07
94	3-Deoxy-arabino-hexonic acid lactone	0.1703	9.08E-10
95	Compound_61	0.14047	1.30E-03
96	Methyl-L-Lysine	0.13914	2.51E-02
97	Asparagine	0.073659	4.23E-02
98	Tartaric acid	0.070333	2.33E-09
99	Cadaverine	0.066192	3.58E-08
100	Uridine	0.055956	3.31E-02
101	Butanedioic acid	0.034119	4.05E-10
102	Aspartic acid	0.030877	3.74E-10
103	Sophorose	0.017878	4.13E-07
104	6-Hydroxymelatonin	0.014419	1.52E-08
